# Screen-based activities and physical complaints among adolescents from the Nordic countries

**DOI:** 10.1186/1471-2458-10-324

**Published:** 2010-06-09

**Authors:** Torbjørn Torsheim, Lilly Eriksson, Christina W Schnohr, Fredrik Hansen, Thoroddur Bjarnason, Raili Välimaa

**Affiliations:** 1 Department of Psychosocial Science, University of Bergen, Bergen, Norway; 2 Swedish National Institute of Health, Östersund, Sweden; 3 Institute of Public Health, University of Copenhagen, Copenhagen, Denmark; 4 Research Centre for Health Promotion, University of Bergen, Bergen, Norway; 5 Faculty of Social Sciences, University of Akureyri, Akureyri, Iceland; 6 Department of Health Sciences, University of Jyväskylä, Jyväskylä, Finland

## Abstract

**Background:**

A positive association between time spent on sedentary screen-based activities and physical complaints has been reported, but the cumulative association between different types of screen-based activities and physical complaints has not been examined thoroughly.

**Methods:**

The cross-sectional association between screen-based activity and physical complaints (backache and headache) among students was examined in a sample of 31022 adolescents from Denmark, Sweden, Norway, Finland, Iceland and Greenland, as part of the Health behaviour in school-aged children 2005/06 (HBSC) study. Daily hours spent on screen-based activities and levels of physical complaints were assessed using self-reports.

**Results:**

Logistic regression analysis indicated that computer use, computer gaming and TV viewing contributed uniquely to prediction of weekly backache and headache. The magnitude of associations was consistent across types of screen based activities, and across gender.

**Conclusion:**

The observed associations indicate that time spent on screen-based activity is a contributing factor to physical complaints among young people, and that effects accumulate across different types of screen-based activities.

## Background

A rising prevalence of physical complaints such as back pain, neck and shoulder pain, and headache has been reported for adolescent populations [1]. Comparing repeated cross sectional surveys from 1991 to 2001, the odds of back pain weekly increased between 23% to 50% for boys and between 44% to 50% among girls across the 10-year period [1]. Parallel to the increase in physical complaints, adolescents spend an increasing amount of time on screen-based activities, such as TV, computer games, or other types of computer based entertainment [2]. These parallel trends might be causally related. A suggested mechanism is that consecutive periods of screen-based activities lead to sustained muscle tension and a lack of recovery from such tension, and subsequently back pain or headache [3]. In line with this hypothesis, excessive screen-based activities have been associated with an increased risk of physical complaints in young people [4-6]. A dosage-response relationship has been documented in some studies [6], while other studies have reported a nonlinear effect [4], or a weak association [7]. If causally related, the adverse public health impact of screen-based activities on the prevalence of physical complaints might be strong as most adolescents indulge in daily screen-based activities [4,8,9].

The extent to which different kinds of screen-based activities are differently related to the risk of physical complaints is not known. Adolescents switch between different types of screen-based activities, e.g. watching TV, playing computer games, or using the computer for other purposes (e.g. internet, chatting, e-mailing, homework etc.). The objective of these activities varies greatly, but a common feature is that the activities usually involve a constant physical position relative to the screen. With increasing screen-based activities across different types of media, it is relevant to address both specific as well as cumulative effects of different kinds of screen-based activities. Such analysis might help to reveal whether involvement in particular activities are more potent risk factors of physical complaints, or whether the relationship generalises, and accumulates across types of activities.

In addressing the role of screen-based activities it is necessary to consider a number of third variables that might affect both screen-based activity and physical health complaints. These include level of physical activity, life stress, and depressive symptoms. The association between time spent on screen-based activities and physical complaints might be spurious due to the parallel inverse association between physical activity and physical complaints. Finally, excessive screen-based activity and somatic complaints might be viewed as causally unrelated markers of depressive symptoms, or a general adaptation to various stressful conditions [10]. Consequently, the association between physical complaints such as backache and headache and screen-based activities might be non-specific.

In relation to screen-based activities among adolescents, it is relevant to analyse health effects of various types of computer use in a large Nordic representative population, since screen-based technology is widespread in these countries. Until now there have been separate studies for Sweden [9] and Finland [4], but no common Nordic study on this relationship. Therefore, the objective of the present study was to assess the independent association between types of screen-based activities and backache and headache in the Nordic countries.

## Methods

The study was based on data from the 2005/06 round of the Health Behaviour in School-Aged Children (HBSC) study, which has been conducted since 1982 [11]. HBSC is an international WHO collaborative study performed every fourth year among 11-, 13- and 15-year-olds, using cross-sectional surveys. The students fill out a standardised questionnaire during a school lesson, after instruction from the teacher. The present study was based on analyses on data from Denmark, Sweden, Finland, Norway, Iceland and Greenland; a total of 31 022 adolescents. The HBSC study uses classes as primary sampling unit. In countries where lists of classes are not available schools are used as the primary sampling unit, with second stage samples of classes within schools. Non-participation might occur at the level of school, school class or individual student. Given the national differences in sampling, it is difficult to calculate an equivalent response rate. A recent review of the methodology of the HBSC study concluded that (p. 143) "...data for 2005/06 suggest that school/class and pupil level response rates exceeded 70% in the majority of countries/regions". Authors' own calculations based on available data suggest that this conclusion also holds for the countries selected for this study. For more information on the HBSC study, see paper by Roberts and colleagues [12]. Necessary ethics approval of the research protocol was secured on a national basis. In some countries ethics approval of the protocol was obtained through a regional or national ethics committee. In Norway the study was approved by the regional ethical committee for health-related research of Western Norway. In Iceland the HBSC project was reported to the government agency Persónuvernd. In Sweden the study was conducted by the state government, whose activity is beyond the responsibility of the regional research ethical committees. In Greenland and in Denmark, there were no central ethical approval, but each school board approved with the study to be able to do the survey in that school. In Finland ethical approval was provided by from National Board of Education in Finland and the Trade Union of Education. Data collection was done during the winter term 2005/06: Sweden (November/December 2005), Norway (December/January), Denmark (February-March), Iceland (February-March), Greenland (March-April) and Finland (March-May).

Adolescents rated their screen-based activity with regard to TV viewing, computer games and computer use, with separate assessment of weekday and weekend frequency. TV viewing was measured by the question: *About how many hours a day do you usually watch television (including videos) in your free time? *Computer gaming was measured asking: *About how many hours a day do you play PC-games or TV-games (Playstation, Xbox, GameCube etc.) in your free time? *Computer use was measured asking: *About how many hours do you spend using a computer (internet, chatting, e-mailing, homework etc..)? *All three questions had the same nine response categories: *None at all, About half an hour a day, About 1 hour a day, About 2 hours a day, About 3 hours a day, About 4 hours a day, About 5 hours a day, About 6 hours a day, About 7 or more hours a day*. For each type of activity a daily average score was computed by weighting weekend and weekday ratings according to the number of days covered (weekday 5/7; weekends 2/7). A recent study evaluated test-retest reliability and relative validity (7-day TV-diary) of the item assessing hours spent watching TV, and no systematic differences were identified between test and retest. However, the reported time adolescents usually spent watching TV was found to exceed the time reported in the TV diary by approximately one hour per day for boys, and half an hour for girls [13].

Subjective physical complaints were assessed by the question: *During the past 6 months, how often have you had the following...? *Below this question a list of eight different physical and emotional complaints was presented, including headache, backache, and feeling low. The five response categories to these eight items were: *About every day, More than once a week, About every week, About every month, Rarely or never*. Qualitative interviews from a validity study [14] indicated that headache and backache often led to the use of medical services. The perceived aetiology was seen as related to lack of activity, type of activity, high workload, and medical illness. A test-retest study showed single item reliability in the range of 0.62 to 0.76, and a summed score reliability of 0.79.

The Family Affluence Scale [15] (FAS II) was used to measure family socioeconomic status. FAS II is a composite score consisting of four items on material assets: *Does your family own a car, van or truck? Do you have your own bedroom for yourself? During the past 12 months, how many times did you travel away on holiday with your family? How many computers do your family own*? FAS has demonstrated good criterion validity across a range of studies [15,16].

Physical activity was measured with an item developed for epidemiological use [17]: *During the past 7 days how many days were you physically active for a total of at least 60 minutes? *A validation study indicated good reliability, and correlation with accelerometer data [17]. School related stress was measured by asking: *How pressured do you feel by the schoolwork you have to do? *Response options were: *Not at all, A little, Some, A lot*. A test-retest study indicated sufficient reliability [18]. In epidemiological studies this indicator has shown strong associations with physical health complaints [19].

All analyses were conducted using Stata 10. The observations were obtained from clustered samples of classes and schools, which might introduce biased standard errors using conventional methods. The magnitude of such bias can be expressed through the design effect (DEFT). Point estimates were obtained using linearised standard errors obtained from Stata SVY module. There was practically no design effect on regression weights (DEFT ranging from 0.99 to 1.05), suggesting ignorable bias in standard errors obtained using conventional maximum likelihood estimators. Thus, to be able to perform nested model comparison in logistic regression analysis, we decided to use conventional maximum likelihood estimators with likelihood ratio test (LRT) of nested model comparisons.

## Results

Table 1 shows the prevalence of backache and headache stratified by gender and country. The proportion of backache and headache differed by country. For boys the percentage with recurrent backache varied from 11% in Greenland to 21% in Iceland. For girls, the prevalence of recurrent backache ranged between 11% in Greenland and 26% in Iceland. For boys, the percentage with recurrent headache differed between 15% in Greenland and 31% in Finland. For girls, the percentage with headache ranged from 26% in Norway to 44% in Finland.

**Table 1:** Physical complaints weekly (%) by country and gender.

	Backache weekly				Headache weekly			
		
	Boys		Girls		Boys		Girls	
				
	Estimate	95% CI	Estimate	95% CI	Estimate	95% CI	Estimate	95% CI
DK	18.2	(16.7 to 19.7)	19.5	(17.9 to 21.2)	16.1	(14.8 to 17.5)	26.3	(24.6 to 28.1)
FI	17.6	(15.9 to 19.5)	19.1	(17.5 to 20.8)	31.0	(29.1 to 32.9)	43.9	(41.8 to 46.1)
GL	11.2	(8.8 to 14.1)	11.1	(8.9 to 13.8)	15.0	(12.5 to 17.8)	26.4	(23.2 to 29.8)
IS	21.5	(20.3 to 22.8)	25.9	(24.5 to 27.5)	27.1	(25.8 to 28.5)	38.1	(36.5 to 39.6)
NO	14.4	(13.0 to 15.9)	18.0	(16.3 to 19.8)	16.3	(14.7 to 18.0)	26.1	(24.2 to 28.1)
SE	16.4	(14.6 to 18.4)	21.6	(19.5 to 23.8)	25.2	(23.2 to 27.2)	39.3	(36.6 to 42.1)
Total	18.0	(17.4 to 18.7)	21.1	(20.4 to 21.9)	23.3	(22.6 to 24.0)	34.8	(33.9 to 35.7)

CI- confidence interval; DK-Denmark; FI-Finland; GL-Greenland; IS-Iceland; NO-Norway; SE-Sweden.

Table 2 shows the mean level of screen-based activities by country and gender. The country differences were generally small. In all countries, TV viewing was the type of activity with the highest mean level. Across countries, computer gaming was the second most prevalent activity for boys, whereas for girls, computer use was the second most prevalent activity. There were marked significant gender differences with regard to computer gaming, but not for computer use and TV viewing.

**Table 2:** Daily hours of screen-based media activity by country and gender

	Daily hours of screen based activity					
	
	Computer use		Computer games		Television viewing	
			
Sample	M	95% CI	M	95% CI	M	95% CI
Denmark						
Boys	1.61	(1.53 to 1.69)	2.28	(2.20 to 2.37)	2.68	(2.61 to 2.75)
Girls	1.45	(1.38 to 1.53)	0.84	(0.78 to 0.90)	2.48	(2.41 to 2.55)
Finland						
Boys	1.54	(1.45 to 1.62)	1.97	(1.88 to 2.06)	2.21	(2.14 to 2.28)
Girls	1.55	(1.48 to 1.62)	0.67	(0.62 to 0.72)	2.13	(2.06 to 2.19)
Greenland						
Boys	1.17	(1.00 to 1.35)	1.70	(1.50 to 1.91)	2.62	(2.40 to 2.83 )
Girls	1.43	(1.23 to 1.63)	0.77	(0.68 to 0.87)	2.60	(2.39 to 2.81)
Iceland						
Boys	1.87	(1.80 to 1.94)	1.99	(1.93 to 2.06)	2.56	(2.51 to 2.61)
Girls	1.72	(1.66 to 1.78)	0.52	(0.49 to 0.55)	2.22	(2.17 to 2.27)
Norway						
Boys	1.58	(1.48 to 1.69)	1.97	(1.88 to 2.07)	2.41	(2.33 to 2.50)
Girls	1.80	(1.69 to 1.90 )	0.71	(0.65 to 0.76)	2.42	(2.33 to 2.51)
Sweden						
Boys	1.75	(1.64 to 1.86)	2.19	(2.08 to 2.30)	2.30	(2.22 to 2.39)
Girls	1.73	(1.62 to 1.84)	0.74	(0.68 to 0.81)	2.17	(2.09 to 2.25)

M-Mean; CI- confidence interval

Table 3 shows the time spent on screen-based activities by status of backache and headache. The table shows that boys with backache weekly reported more time watching TV, more time playing computer games, and more time using computers. Girls with either backache or headache tended to spend more time watching TV and computers, but did not spend more time on computer games, compared to girls without physical complaints.

**Table 3:** Daily hours spent on screen-based media by symptom status.

	Daily hours of screen based activity					
	
	Computer use		Computer games		Television viewing	
			
Physical symptom status	M	95% CI	M	95% CI	M	95% CI
Boys						
Backache						
Less than weekly	1.60	(1.56 to 1.63)	1.99	(1.95 to 2.03)	2.41	(2.38 to 2.44)
Weekly	2.13	(2.05 to 2.21)	2.40	(2.31 to 2.49)	2.72	(2.65 to 2.79)
Headache						
Less than weekly	1.61	(1.57 to 1.65)	1.99	(1.95 to 2.04)	2.42	(2.39 to 2.46)
Weekly	1.95	(1.88 to 2.02)	2.29	(2.21 to 2.36)	2.60	(2.54 to 2.66)
Girls						
Backache						
Less than weekly	1.54	(1.50 to 1.57)	0.66	(0.63 to 0.68)	2.22	(2.19 to 2.25)
Weekly	2.03	(1.96 to 2.09)	0.73	(0.69 to 0.78)	2.52	(2.46 to 2.58)
Headache						
Less than weekly	1.48	(1.44 to 1.52)	0.66	(0.63 to 0.68)	2.21	(2.17 to 2.24)
Weekly	1.94	(1.89 to 1.99)	0.70	(0.66 to 0.74)	2.42	(2.38 to 2.47)

M-Mean; CI-confidence interval

Table 4 shows the polyserial correlation matrix for the study variables. In general, there was a weak to moderate correlation between types of screen-based activity. Potential third variables, such as physical activity and school-related stress correlated weakly with physical complaints and with screen-based activities. Feeling low correlated moderately with physical complaints.

**Table 4:** Polyserial correlation between study variables, pooled analysis.

	**1**.	**2**.	**3**.	**4**.	**5**.	**6**.	**7**.	**8**.
1. Backache weekly		0.43	0.42	-0.10	0.28	0.04	0.18	0.13
2. Headache weekly	0.39		0.42	-0.08	0.28	0.03	0.19	0.10
3. Feeling low weekly	0.38	0.39		-0.14	0.37	0.06	0.23	0.11
4. Moderate physical activity	-0.08	-0.07	-0.13		-0.12	-0.05	-0.13	-0.16
5. School-related stress	0.25	0.22	0.28	-0.09		-0.02	0.19	0.10
6. Computer games	0.09	0.07	0.05	-0.14	0.06		0.28	0.26
7. Computer use	0.15	0.10	0.10	-0.10	0.13	0.35		0.28
8. Television viewing	0.11	0.07	0.06	-0.10	0.08	0.35	0.27	

Boys below the diagonal, girls above the diagonal.

Table 5 shows pooled analysis odds-ratio of backache and headache per added daily hour of screen-based activity, with mutually adjusted estimation for TV viewing, computer gaming, and computer use. Country was modelled as a fixed main effect. For both genders, there was a statistically significant increase in backache per hour spent on watching TV, using the computer, and playing computer games. For each hour spent on computers a boy and a girl would increase the odds of backache by 8% and 10%, respectively.

**Table 5:** Pooled analysis of physical complaints regressed on computer use, computer gaming and TV-viewing.

	M1:Mutually adjusted		M2:Mutually adjusted + adjusted for confounders	
		
	OR	95%CI	OR	95%CI
	Dependent: Backache weekly			
Boys				
Computer use	1.08	(1.06 to 1.11)	1.06	(1.03 to 1.09)
Computer games	1.05	(1.02 to 1.08)	1.04	(1.01 to 1.07)
Television viewing	1.06	(1.03 to 1.09)	1.05	(1.02 to 1.09)
Girls				
Computer use	1.10	(1.07 to 1.14)	1.05	(1.02 to 1.08)
Computer games	1.06	(1.02 to 1.10)	1.05	(1.01 to 1.10)
Television viewing	1.09	(1.05 to 1.12)	1.08	(1.05 to 1.12)
	Dependent: Headache weekly			
Boys				
Computer use	1.06	(1.03 to 1.09)	1.04	(1.01 to 1.06)
Computer games	1.04	(1.02 to 1.07)	1.03	(1.01 to 1.06)
Television viewing	1.05	(1.02 to 1.08)	1.04	(1.01 to 1.07)
Girls				
Computer use	1.15	(1.12 to 1.18)	1.10	(1.07 to 1.13)
Computer games	1.00	(0.97 to 1.04)	0.99	(0.96 to 1.03)
Television viewing	1.08	(1.05 to 1.11)	1.07	(1.04 to 1.10)

OR - odds ratio; CI - confidence interval. Screen-based activity time was included in the logistic regression models as a continuous variable. In M1 ORs were adjusted for country, age, and socio-economic status. In M2 ORs were also adjusted for depressed mood, school-related stress and physical activity.

Boys' computer use, computer gaming and TV viewing were associated with increased odds of headache. Girls' computer use and TV-viewing, but not computer gaming, were associated with increased odds of headache.

To examine whether the association between screen-based activities and physical complaints was homogeneous across countries, country by screen-based activity interaction effects were included to the model. This interaction term was not statistically significant. Grade by screen time interaction on headache was not statistically significant (Boys: LRT Chi-square test (6) = 10.89; Girls: LRT Chi-square test (6) = 5.98). For boys, but not for girls, there was a statistically significant grade by screen time interaction on backache (Boys: LRT Chi-square test (6) = 18.17, p < 0.01; Girls: LRT Chi-square difference test (6) = 10.74).

To examine nonlinear effects, the squared product term of each screen-based activity was added to the main effect. There were no consistent evidence of quadratic nonlinear effects on backache and headache. However, an omnibus test of nonlinearity on 3 degrees of freedom indicated a nonlinear effect for boys on headache (LRT Chi-square difference test (3) = 9.31, p < 0.05) and on backache (LRT Chi-square difference test (3) = 11.77, p < 0.01). The main source of nonlinearity for boys was a progressively increasing effect of TV viewing on backache, and a progressively diminishing effect of computer use on headache. For girls, quadratic nonlinear effects were not statistically significant.

## Discussion

The study found a weak but consistent association between screen-based activities and odds of recurrent backache and headache among Nordic adolescents. A particular contribution from the present study was that the effects of different types of screen-based behaviours were estimated in the same model, and mutually adjusted. After mutual adjustment, TV viewing, computer use and computer gaming showed unique associations with backache and headache. This pattern suggested little evidence of differential association with physical complaints. A notable exception to this pattern was the association between screen-based activity and headache in girls. In girls, computer use and TV viewing, but not computer gaming, increased the odds of headache.

Overall, the consistent association between screen-based activity and physical complaints might indicate that a part of the association is unrelated to the type of screen-based activity, but rather more related to the duration and ergonomic aspects of such activity. Unfortunately, the current study design did not enable measurement of ergonomic aspects of the activities. However, if the content of the activity were a key factor, a stronger differentiation between the types of activity would be expected.

The findings are in line with a recent study of Finnish adolescents [4] in which computer use and watching TV were associated with neck and shoulder pain and lower back pain. The results point to time spent on screen-based activities as a specific risk factor, but the relatively weak associations suggest that screen-based activities are contributors to, but not sufficient or necessary causes of physical complaints. In contrast to other studies [4], we found no support for a nonlinear threshold effect of health complaints, even though the statistical power of the present study was high. Rather, the results of this study indicated a monotonous increase in backache and headache with increasing screen time, and were thus in line with the findings of Smith and colleagues [6]. This pattern indicated that it is difficult to establish a cut-off that discriminates between harmful and non-harmful time spent on screen-based activities.

In support of the internal validity of the study, the associations between screen-based activities and physical complaints remained after controlling for confounding influences. First, the associations remained intact after controlling for physical activity, indicating that a lack of physical activity cannot explain the association between screen-based activity and physical complaints. Secondly, although it has been suggested that depressed mood and anxiety lower the threshold for pain experience, and increase the risk for sedentary behaviour, the observed association remained intact after adjusting for depressed mood, suggesting that the association was not spurious due to common influence of depressed mood. Finally, controlling for school-related stress did not alter the associations. The minor reduction of effect sizes after adjustment for potential confounders indicated that a significant proportion of the relationship between physical complaints and screen-based activities was specific to the amount of time spent on such activities.

Although most adolescents engage in screen-based activity, relatively few individuals report excessive use, typically defined as five or more hours a day. Thus, the precision of estimates for individuals with very high use tends to be low in most studies. A strength of this study is the sample of 31 022 adolescents collected in the Nordic countries, ensuring good coverage even in the higher range of screen-based activity. The study adds to the field of health effects from screen-based activities by providing relatively stable estimates due to the large sample size, and the knowledge that there are only small variations between the countries under study of the determinants and outcomes.

Limitations of the present study include cross-sectional design and self-reported screen-time. The cross-sectional study design offers a weak basis for examining the direction of effects. For example, one could argue that adolescents with backache and headache are less able to indulge in physical activities, and thus spend more time in front of the TV or computer. However, according to principles of mediation we would then expect no association between screen time and physical complaints after adjusting for physical activity.

The second limitation is self-reported screen-time, which might introduce recall bias. Importantly the measurements of screen-based activity are not mutually exclusive, and adolescents' activity might overlap across media types. For example, adolescents might use a laptop computer and watch TV at the same time, or even watch TV on their computer. Such overlap indicates that one should avoid adding the reports across indicators to achieve a total screen time, since such addition would likely overestimate the total screen time. This pattern strengthens the need for mutually adjusted estimates, and supports the strategy chosen in the present study.

## Conclusions

The results indicate that time spent on screen based activities is a potential contributing factor to physical health complaints. The HBSC study will continue to monitor the cross-national development in the Nordic countries in the coming years, but longitudinal studies are needed to examine the causal relation between screen-based activities and health complaints.

## Competing interests

The authors declare that they have no competing interests.

## Authors' contributions

TT conceptualised the paper, conducted analysis, and wrote the text. LE, CWS and FH wrote text and provided feedback on the paper. TB and RV read and contributed with revising the paper. All authors read and approved the final manuscript.

## Pre-publication history

The pre-publication history for this paper can be accessed here:

http://www.biomedcentral.com/1471-2458/10/324/prepub

## References

[B1] Hakala P Rimpela A Salminen JJ Virtanen SM Rimpela M Back, neck, and shoulder pain in Finnish adolescents: national cross sectional surveys British Medical Journal 2002 325 7367 743 745 10.1136/bmj.325.7367.743 12364301 PMC128374

[B2] Nelson MC Neumark-Stzainer D Hannan PJ Sirard JR Story M Longitudinal and secular trends in physical activity and sedentary behavior during adolescence Pediatrics 2006 118 6 E1627 E1634 10.1542/peds.2006-0926 17142492

[B3] Straker LM Coleman J Skoss R Maslen BA Burgess-Limerick R Pollock CM A comparison of posture and muscle activity during tablet computer, desktop computer and paper use by young children Ergonomics 2008 51 4 540 555 10.1080/00140130701711000 18357540

[B4] Hakala PT Rimpela AH Saarni LA Salminen JJ Frequent computer-related activities increase the risk of neck-shoulder and low back pain in adolescents European Journal of Public Health 2006 16 5 536 541 10.1093/eurpub/ckl025 16524936

[B5] Sjolie AN Associations between activities and low back pain in adolescents Scandinavian Journal of Medicine & Science in Sports 2004 14 6 352 359 10.1111/j.1600-0838.2004.377.x 15546330

[B6] Smith L Louw Q Crous L Grimmer-Somers K Prevalence of neck pain and headaches: impact of computer use and other associative factors Cephalalgia 2009 29 2 250 257 10.1111/j.1468-2982.2008.01714.x 19143770

[B7] Diepenmaat ACM van der Wal MF de Vet HCW Hirasing RA Neck/Shoulder, Low Back, and Arm Pain in Relation to Computer Use, Physical Activity, Stress, and Depression Among Dutch Adolescents Pediatrics 2006 117 2 412 416 10.1542/peds.2004-2766 16452360

[B8] Marshall SJ Gorely T Biddle SJH A descriptive epidemiology of screen-based media use in youth: A review and critique Journal of Adolescence 2006 29 3 333 349 10.1016/j.adolescence.2005.08.016 16246411

[B9] Palm P Risberg EH Mortimer M Palmerud G Toomingas A Tornqvist EW Computer use, neck and upper-extremity symptoms, eyestrain and headache among female and male upper secondary school students Scandinavian Journal of Work Environment & Health 2007 33 41

[B10] van den Eijnden R Meerkerk GJ Vermulst AA Spijkerman R Engels R Online communication, compulsive Internet use, and psychosocial well-being among adolescents: A longitudinal study Developmental Psychology 2008 44 3 655 665 10.1037/0012-1649.44.3.655 18473634

[B11] Currie C Nic Gabhainn S Godeau E the International HNCC The Health Behaviour in School-aged Children: WHO Collaborative Cross-National (HBSC) Study: origins, concept, history and development 1982-2008 International Journal of Public Health 2009 54 0 131 139 10.1007/s00038-009-5404-x 19639260

[B12] Roberts C Freeman J Samdal O Schnohr C de Looze M Nic Gabhainn S Iannotti R Rasmussen M the International HSG The Health Behaviour in School-aged Children (HBSC) study: methodological developments and current tensions International Journal of Public Health 2009 54 0 140 150 10.1007/s00038-009-5405-9 19639259 PMC2732766

[B13] Vereecken CA Todd J Roberts C Mulvihill C Maes L Television viewing behaviour and associations with food habits in different countries Public Health Nutrition 2006 9 2 244 250 10.1079/PHN2005847 16571179

[B14] Haugland S Wold B Subjective health complaints in adolescence - Reliability and validity of survey methods Journal of Adolescence 2001 24 5 611 624 10.1006/jado.2000.0393 11676508

[B15] Currie C Molcho M Boyce W Holstein B Torsheim T Richter M Researching health inequalities in adolescents: The development of the Health Behaviour in School-Aged Children (HBSC) Family Affluence Scale Social Science & Medicine 2008 66 6 1429 1436 10.1016/j.socscimed.2007.11.024 18179852

[B16] Andersen A Krolner R Currie C Dallago L Due P Richter M Okenyi A Holstein BE High agreement on family affluence between children's and parents' reports: international study of 11-year-old children Journal of Epidemiology and Community Health 2008 62 12 1092 1094 10.1136/jech.2007.065169 18413436

[B17] Prochaska JJ Sallis JF Long B A physical activity screening measure for use with adolescents in primary care Archives of Pediatrics & Adolescent Medicine 2001 155 5 554 559 11343497 10.1001/archpedi.155.5.554

[B18] Torsheim T Wold B Samdal O Haugland S Test-retest reliability of survey indicators measuring adolescent health and health behaviour 1997 University of Bergen: Research Centre for Health Promotion

[B19] Torsheim T Wold B School-related stress, school support, and somatic complaints: A general population study Journal of Adolescent Research 2001 16 3 293 303 10.1177/0743558401163003

